# An Autonomous Wireless Device for Real-Time Monitoring of Water Needs

**DOI:** 10.3390/s20072078

**Published:** 2020-04-07

**Authors:** Juan D. Borrero, Alberto Zabalo

**Affiliations:** 1 Department of Business and Marketing, Huelva University, Huelva 21071, Spain; 2 Department of Agroforestry Science, Huelva University, Huelva 21071, Spain; alberto.zabalo@dcaf.uhu.es

**Keywords:** LoraWAN, Internet of Things (IoT), agriculture 4.0, wireless sensor network, energy consumption

## Abstract

The agri-food sector is in constantly renewing, continuously demanding new systems that facilitate farmers´ work. Efficient agricultural practices are essential to increasing farm profitability, and reducing water consumption can be achieved by real-time monitoring of water needs. However, the prices of automatic systems for collecting data from several sources (soil and climate) are expensive and their autonomy is very low. This paper presents a low-consumption solution using the Internet of Things (IoT) based on wireless sensor networks (WSNs) and long-range wide-area network (LoRaWAN) technologies. By means of low-power wide-area network (LPWAN) communication, a farmer can monitor the state of crops in real time thanks to a large number of sensors connected wirelessly and distributed across the farm. The wireless sensor node developed, called BoXmote, exhibits very low power, since it has been optimized both in terms of hardware and software. The result is a higher degree of autonomy than commercial motes. This will allow the farmer to have access to all of the information necessary to achieve an efficient irrigation management of his crops with full autonomy.

## 1. Introduction

Some of the main problems that the world population faces today, and on which researchers from all over the world are working, are globalization, global warming, and the supply of food. In order to address the latter, it is necessary to develop modern solutions that allow the optimization of costs and increased yields, such as the smart management of water for precision irrigation in agriculture [1,2].

In recent years, many researchers have been integrating the concept of the Internet of Things (IoT) into the field of agriculture, thus helping the development of what is known as precision agriculture. Precision agriculture aims to provide a decision support system that helps farmers to implement efficient agricultural practices, with the aim of increasing profitability, reducing environmental risks, and preserving natural resources [3,4].

So-called smart agriculture or digital agriculture uses intelligent networks and data management tools to employ all of the information and experience available in order to enable the automation of sustainable processes in agriculture. This agriculture is based on connected and knowledge-based agricultural production systems and makes great use of information and communication technologies (ICT), both for data collection and for subsequent processing. For this reason, the design and implementation of efficient and economic devices in agriculture are currently an important line of research [5].

Due to the characteristics of the agricultural sector, the use of wired systems for the collection of information is practically unfeasible, which is why work is currently underway to develop wireless sensor networks (WSNs) in order to minimize the economic cost and impact of installation in agricultural environments, thus avoiding hindering the work of the farmer [5,6].

WSN solutions for establishing different parameters in agriculture should include certain requirements for smaller farmers to implement this type of system on their farms, such as inexpensive devices (i.e., the cost of a radio chipset being less than 2€ and an operating cost of 1€ per device per year), low energy consumption (i.e., 10+ years of battery lifetime), and a long range of communication (i.e., up to 10-40 km in rural zones)—three areas in which we must work in the future [2,7,8].

Currently, there are some challenges to be overcome that still prevent the use of IoT for irrigation. Firstly, there are still many rural areas that do not have Internet connectivity. Secondly, they do not have electricity either. Thirdly, monitoring with soil sensors requires scalability, but this comes at a price. Finally, the integration of different and advanced sensors requires adequate standards. In order to solve the aforementioned problems, this paper develops and assesses an inexpensive WSN solution based on long-range (LoRa) technology with very low power consumption and high autonomy to control water needs for precision irrigation in agriculture.

Many additional mechanisms have been proposed to increase the battery life of WSN end-devices and to accomplish energy saving, irrespective of whether the devices are already of low consumption. However, we found it difficult to compare them due to the diversity of the methodologies, end-device components, sensors used, and types of experiment.

Therefore, the main contribution is that due to the fact that existing WSN systems for precision irrigation are mostly theoretical with limited proof of concept experiences in practice, providing details of devices and equipment as well as irrigation techniques [2,4], this work compares consumption to show a more energy-efficient end-device configuration.

## 2. Wireless Sensor Networks in the Context of Smart Agriculture

We can see different solutions for monitoring crops using WSNs. Many of these proposals have only been tested in the laboratory and still have to be developed and implemented on real crops.

A low-cost, small-scale system to remotely monitor and control a greenhouse was created in [9]. In this study, the information was collected by an Arduino Mega and sent to a web page through ethernet communication.

In [10], a system based on short message service (SMS) communication using a global system for mobile (GSM) module was developed to measure parameters such as soil moisture, pH, and atmospheric pressure. With these data, irrigation could be controlled and the farmer informed of the state of his crops. Similarly, the authors of [11] developed a WSN system with a soil moisture sensor and a temperature and humidity sensor.

One of the most widely used technologies for creating wireless sensor networks in agriculture is the ZigBee technology. In [5,12,13], a WSN was presented for monitoring crops by measuring parameters such as soil moisture, pH, conductivity, salinity, and ambient light.

General packet radio service (GPRS) technology can also be used in agriculture to create WSNs [6]. In [14], the authors developed an automated irrigation system to optimize the use of water in crops by combining the ZigBee and GPRS technologies. By means of this system, the humidity and temperature of the land can be measured. Furthermore, in [15], an IoT-based irrigation system was presented using soil moisture sensors controlled by ATMEGA 328P on an Arduino UNO board, along with a GPRS module.

NarrowBand IoT (NB-IoT) is an LPWAN technology based on narrow band radio technology that can coexist with GSM and long-term evolution (LTE) under licensed frequency bands. NB-IoT is standardized by the 3rd Generation Partnership Project (3GPP) and its specifications were released in June 2016; thus, additional time is needed before its network is established [16].

SigFox is a new technology used in IoT applications. In [17], a geolocation system was developed to locate herds of cattle. As advantages of the SigFox technology, we can highlight its wide range of coverage and low consumption, which makes it very appropriate for IoT solutions.

With SigFox, LoRa technology has started to be used in precision agriculture in recent years as an alternative to previous protocols [18,19]. It is a long-range and low-power wireless communication system whose objective is to be used in long-term devices [20]. Based on the LoRaWAN technology, the MoniSen end-device [1] used the ATMEGA324P processor and the RN2473 chip to establish LoRa communication, to measure parameters such as ambient temperature and humidity, soil temperature and humidity, and light intensity received by the plant. In a smart irrigation system [19], the end-device achieved a distance of 7 km in the field tests.

Table 1 shows a comparison of the different communication technologies discussed above for the creation of WSNs [8,16,21,22].

If we analyze the data shown in the table above, we can see that GPRS technology, apart from its high cost, is not entirely suitable for the communication of wireless sensors due to its high energy consumption, which means it is little used in the creation of high autonomy nodes.

Currently, the ZigBee wireless communication protocol is considered one of the best technologies in precision agriculture, given its low consumption. If we compare it with the LoRa and Sigfox technologies, we can see that all three have low consumption and low cost, making them ideal for the creation of WSNs. However, one of its main disadvantages is its short reach between nodes—100 m in open spaces, as shown in Table 1—which is both lower than the other wireless communication protocols and a great limitation for agriculture.

NB-IoT is preferred for applications that require guaranteed quality of service, whereas applications that do not have this constraint could choose both LoRa or Sigfox. Furthermore, the additional energy consumption because of synchronous communication reduces NB-IoT’s end-device lifetime as compared to them. In addition, NB-IoT is not suitable for rural areas that do not benefit from LTE coverage. Thus, for applications that do not have large amount of data to send, Sigfox and Lora are the best options [8,16].

In the field of agriculture, a long battery lifetime of sensor devices is required. Using temperature, humidity, and electrical conductivity (EC), sensors could significantly reduce water consumption and improve yield, as discussed in [23]. Devices update sensed data a few times per hour, as the environment conditions do not radically change in this time period. Thus, Sigfox and LoRaWAN are ideal for this application. Moreover, various farms do not have LTE cellular coverage, thus NB-IoT is not a solution for agriculture in the near future.

Although the LoRa and SigFox technologies have a great technical similarity, LoRa technology was used in this work for two reasons: Firstly, because we wanted to build a WSN that avoided dependence on third parties. One of the main features of this technology is that any user can build their own network, thus avoiding having to subscribe in order to use an external network. Secondly, because with LoRa, we can support coverage in remote rural areas.

Finally, commercial wireless detection modules such as eKo Pro [24], MicaZ [25], TelosB [26], Zolertia [27], Adcon Telemetry GmbH [28], AquacomSensor Hub Aquaflex [29], Enviroscan [30], or Waspmote [31] for agriculture and its irrigation are still very expensive—usually exceeding 1000.00€—making it impossible for smaller farms to implement this type of system, as well as for smart farms, which require a large number of wireless end-devices for data collection that support reliable decision making.

## 3. Materials and Methods

In the first instance, we installed the LoRa gateway in an elevated position [32], in the headquarters of an agricultural cooperative from which coverage of nearby crops could be given. Thanks to the LoRa technology, we could easily cover an area of about 5 km around this cooperative; this is less than other studies, but we did not modify the LoRaWAN PHY layer and did not point the antenna toward the test area [32,33]. Then, end-devices (BoXmotes, Bo True Activities SL, Huelva, Spain) were installed in the crop, which were equipped with the most suitable sensors, depending on the parameters that were to be measured. In this way, the BoXmote took measurements at regular intervals and sent them to the gateway through the LoRa technology. Once the gateway received the data, they were sent to a server via the ethernet connection. When the data reached the server, the farmer could consult them through a mobile application.

### 3.1. Hardware Description

In this section, a complete description of the hardware that makes up our end-device is given. When choosing the hardware that forms our end-device, it should be noted that all of the components were selected with low consumption in mind, thus increasing battery life to the maximum.

Each end-device comprised a radio transceiver, a micro-controller, sensors, and an antenna, along with other circuitry that would enable it to communicate with the gateway to transmit information collected by the sensors [34].

#### 3.1.1. Monitored Parameters and Sensors

According to [4], the majority of papers monitored between three and four parameters with soil moisture being the most used in soil sensors, and air temperature and humidity as atmospheric parameters. Soil temperature, electrical conductivity (EC), and luminosity are other parameters that are measured regularly [5].

In relation to the measurement of soil moisture, most studies use the conductivity between two electrodes that are inserted into the soil by measuring the constant dielectric of the soil. Most published works use the low-cost sensors YL69 (Sparkfun Electronics, Niwot, CO, USA) and VH400 (Vegetronix, Inc., Riverton, UT, USA) that operate with Arduino. However, we both tested and discarded them because they did not behave stably under real conditions.

Thus, to measure the soil parameters, we used well-known commercial sensors [35] (see Table 2) as the capacitive sensor of the frequency domain reflectrometry (FDR) type ECH2O 10HS (Meter Group, Inc, Pullman, WA, USA) to measure soil moisture, and ECH2O 5TE to measure soil moisture and temperature, as well as EC. These sensors were not specifically created to be integrated in an Arduino measuring system, but we developed our own code written in the open source Arduino Integrated Development Environment (IDE) software and uploaded it to the board to be able to work with it.

The low-cost DHT11/DHT22 sensors (Adafruit Industries, New York, NY, USA), which provide temperature and relative humidity readings, as well as light-dependent resistance (LDR), are the most commonly used sensors for smart irrigation monitoring [4]. Just as we did to test low-cost soil sensors, we tested these sensors for a year in real conditions and they worked well, and since IoT irrigation systems generally do not need the highest level of accuracy, we decided to use them for our work.

#### 3.1.2. LoRa Technology

LoRa, whose meaning is “long range”, is a long-range communications system promoted by the LoRa Alliance [36]. This system is aimed to be used in devices where energy consumption is of the utmost importance.

LoRa can commonly refer to two distinct layers [20]:

1. A physical (PHY) layer, the so-called LoRa, that uses the radio modulation technique chirp spread spectrum (CSS).
2. A medium access protocol (MAC) layer in which LoRaWAN defines the communication protocol and the specific access network architecture.

The LoRa modulation physical layer (Semtech Camarillo, CA, USA) allows a wide range at low power. We used the RN2483 transceiver module for Lora Class A, which operates in the industrial, scientific, and medical (ISM) frequency band of 868 MHz. The payload of each transmission can vary from 2 to 255 octets and the data rate can reach 50 Kbps [1].

LoRaWAN provides a MAC mechanism, which allows many end-devices (end-nodes) to communicate with a single gateway using LoRa modulation. A typical LoRa network shows a star-of-stars topology, which includes three different types of devices—the end-devices, the LoRaWAN gateways, and the network service [20,32].

**Figure 1:**
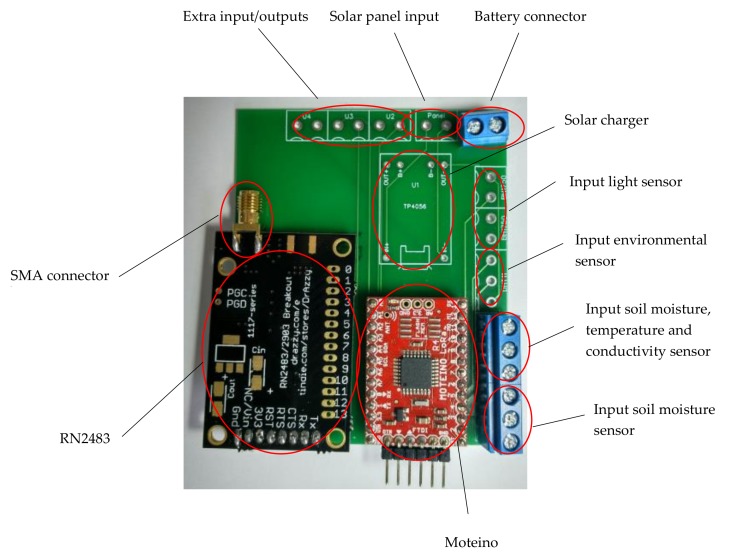
Photograph of the wireless sensor node.

In the basic architecture of a LoRaWAN network (Figure 1), the end-nodes communicate with the corresponding gateway using LoRa with a LoRaWAN link, and the gateways send LoRaWAN frames to a network server via a broadband link (i.e., Ethernet or GPRS) [20].

#### 3.1.3. Sensor Node

The node is the device in charge of collecting the necessary data in the field to later transmit to the gateway. Its two main characteristics must be low consumption and being able to establish communication at the greatest possible distance.

Arduino boards (Smart Projects Srl, Scarmegno, Italy), such as Arduino UNO and Arduino Mega, are the most utilized nodes for the implementation of irrigation systems, and the most utilized controller is the ATmega328 (Atmel, San José, CA, USA) [4].

The selection of the best node for an IoT irrigation system depends on the necessities and the characteristics. We opted for developing our own design for the nodes, with the aim of addressing our own particular requirements of low power consumption and autonomy.

Our end-node includes a processor fully compatible with Arduino IDE. Moteino (LowPowerLab, LLC, Michigan, MI, USA) is ideal for IoT applications because of its very small size (1.3 × 0.9 inch). The moteino used is a versatile low-cost and low-power Arduino based on the Atmel ATMega328P microcontroller. Moteino gives years of run time at a low transmission rate, with a 2000–2600 mAh battery. During sleep mode the radio power consumption is about 0.1 uA; during receive mode around 15mA; and in transmit mode the power consumption is up to 130 mA.

On the other hand, to establish LoRa communication with the gateway, the node has the RN2483 chip. This chip was also optimized to obtain the lowest possible consumption, in addition to being certified by the LoRa alliance. As small farmers cannot afford commercial solutions, we designed a shield for implementing the low-cost IoT irrigation system. This system can integrate numerous sensors that measure a large number of agricultural parameters, both soil and atmospheric (See Figure 1).

A lithium-ion polymer (LiPo) battery with a nominal voltage of 3.7 V and a capacity of 2000 mAh, equipped with a protection circuit that prevents overcharging as well as deep discharging, was used to power the node. As an alternative, Li-Ion batteries with 3.7 V nominal voltage and a capacity of 2600 mAh can also be used, obtaining greater autonomy. LiPo batteries were chosen as they can achieve a high load density with a very small weight and size. Additionally, with the implementation of a small solar panel and the TP4056 module, the battery could be recharged continuously, increasing the device´s autonomy and providing the discharge effect.

In order to guarantee correct communication, the node was equipped with an omnidirectional antenna with SubMiniature version A (SMA) connector with an operating frequency of 868 MHz.

Finally, all of the components were placed in a polyester enclosure (IP66) with a silicone lid seal, in which all of the necessary holes were drilled to mount the connections of the antenna and the sensors. To connect the (external) sensors to the (internal) electronics, we used cable glands (IP68) (Bopla Gehäuse Systeme GmbH, Bünde, Germany) for the BoXmote placed in the field test.

**Figure 2:**
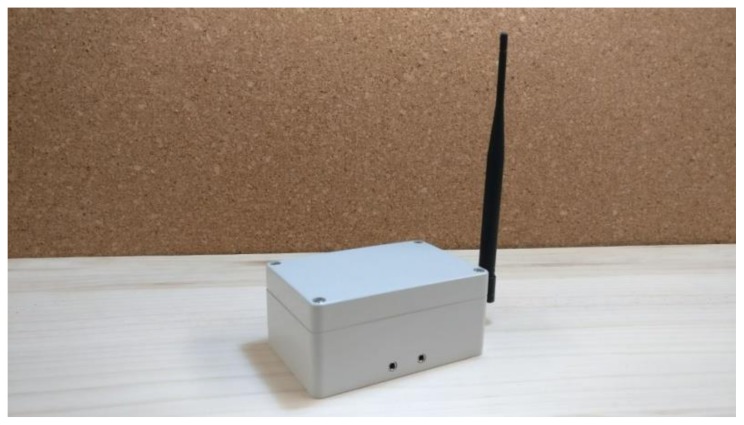
Node sensor (end-device for the lab test).

In the following image (Figure 2), the completely assembled node (end-device for the lab test) is shown.

#### 3.1.4. Gateway

The Gateway is the element in charge of receiving the information from the nodes and taking it to the network. It consists of a microprocessor, a concentrator, and an antenna.

First, the antenna receives the information sent by the node and communicates it to a Raspberry Pi 3 Model B+ (Sony, Pencoed, Wales) microprocessor through the IC880a concentrator, which includes a Semtech SX1301 chip.

The base-station placements were at the edge of the rooftop on a building in the University of Huelva for the laboratory test, and on the rooftop of a cooperative for the field test. The base-stations employed in our experiments were ISM-868MHz outdoor omnidirectional antennas (SIRIO GP 868 C and GP901C models).

Then, the Raspberry Pi is responsible for processing all of the information necessary to upload the information to the network through the previously installed software.

In this work, we estimated the consumption in a rural scenario using fixed positions from the end-device to the gateway, not under mobility conditions [32].

### 3.2. Software Description

Since the Moteino driver is fully compatible with Arduino, it was programmed using the Arduino IDE software with the C++ programming language.

**Figure 3:**
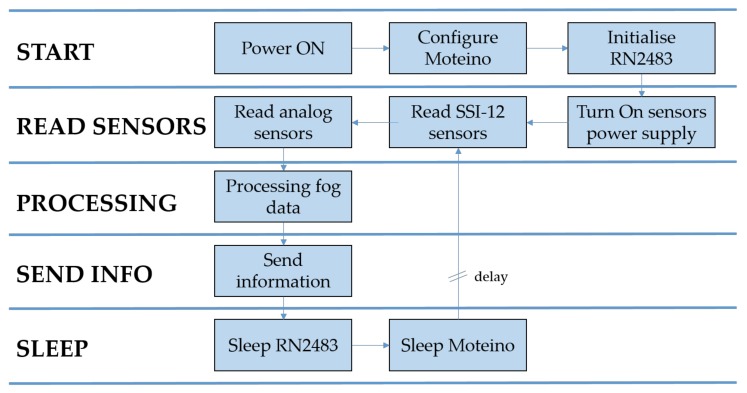
Flow chart of the node sensors.

The software operation of the node is shown in the flow diagram (Figure 3). When the device is activated, the different outputs and inputs of the processor are configured. Next, the RN2483 chip is initialized, through which the information is later transmitted. Then, the necessary data are collected from the different sensors that are connected to the node at that moment. Since the sensors deliver a specific voltage depending on the physical parameter we wish to measure, it is necessary to transform this reading into specific physical units. To this end, data processing is carried out following the guidelines set by the manufacturers. To save battery, only the necessary data are processed (fog computing). The rest is processed in the cloud. Once the desired data are obtained, these are sent through the RN2483 module to the nearest gateway.

After having sent the information, the LoRa module enters a “sleep” mode, through which it is possible to lower its consumption drastically. Then, the same is done with the Moteino microcontroller. In this way, the device has minimum consumption during the time between measurements, preserving the battery life well.

The time between measurements must be programmed to extend the battery life, but also, in turn, to have sufficient precision to quickly detect changes in soil moisture or weather conditions, and it is not necessary to monitor the parameters in real-time [4]. In our experience, for the cultivation of berries in hydroponics, taking measurements every 30 min is sufficient. In irrigated systems with trees, they tend to be taken with less frequency, as well as in outdoor irrigated crops too. Thus, we used the most extreme case.

## 4. Results

As already mentioned, one of the main characteristics that an IoT device must have is low consumption. Specifically, in the agriculture sector, it is essential to design a very low consumption device with which a great autonomy is achieved [37]. In this way, the farmer does not have to worry about the maintenance of the device during the months of maximum work, being able to devote his time to other tasks. Given the importance of a high autonomy of the device in agriculture, in this study we focused on quantifying the consumption of our BoXmote, performing a consumption test in the laboratory. Then, from the data obtained in the said test, the life of the battery in a real crop was estimated. Finally, a comparison of the results obtained with other existing devices was made.

For the experiments, we followed the methodology used in [1,6,13,38]. The node used to perform the experiment consisted of a Moteino microcontroller and a LoRa module with the RN2483 chip. To measure volumetric water content, soil temperature, and bulk electrical conductivity data, it was decided to use ECHOH2O 5TE and ECHOH2O 10HS sensors, which are widely used in agriculture. As regards the battery, a lithium battery with a nominal voltage of 3.7 V and a capacity of 2000 mAh was available.

To perform the experiment, the node was configured to take measurements every 93 s. It was decided to perform the test with such a short interval between measurements in order to decrease the total duration of the experiment. In order to save the battery as much as possible in the intervals in which the node is not working, the different components go to sleep mode, drastically lowering the consumption and preserving even more the battery charge.

The node was located in the laboratory at a temperature of approximately 20 °C with the meter sensors in the air, and at an approximate distance between the node and the Gateway of 600 m.

In order to correctly evaluate the consumption of the device, its process was divided into three states:

- Active mode: in this mode, the node configures the microcontroller and initializes the RN2483 chip. Once these actions have been carried out, it proceeds to read the data provided by the sensors and performs the subsequent processing of the information.
- Send mode: this mode of operation is the one that consumes the most energy, since it is the moment in which the information is sent to the nearest gateway.
- “Sleep” mode: Unlike in the previous case, this mode saves the maximum battery charge as it is not necessary to take or transmit measurements. To do this, both the microcontroller and the RN2483 chip “fall asleep.”

**Figure 4:**
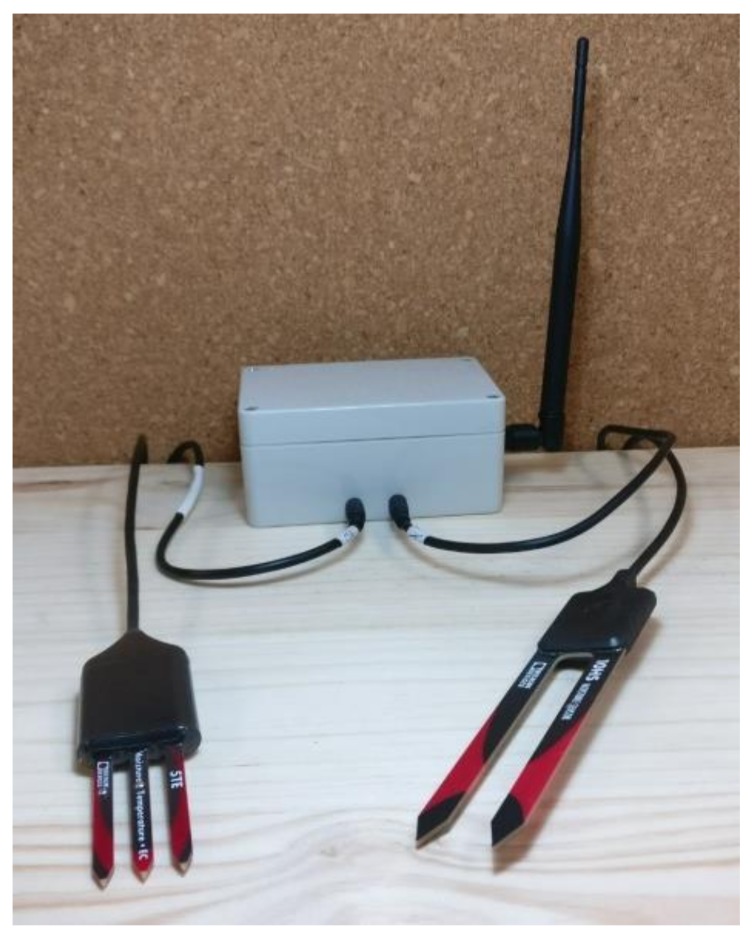
Node with ECH2O soil moisture sensors.

Figure 4 shows the BoXmote with which the battery consumption test was carried out. As previously mentioned, the 5TE and 10HS sensors were incorporated, since they are currently the most widely used in agriculture.

### 4.1. Experimental Laboratory Setup

In the first experiment, real measurements of consumption were taken in the different modes of operation, as well as the time that each one remains active. We used a Digital Multimeter JHS MY-64 [39] to realize the current measurement. To ascertain the state of the end-device, we added a light-emitting diode (LED, sleep mode indicator) to monitor it. The consumption shown takes all the parts of the node (antenna, all sensors, processing, etc.) into consideration. The measurements were performed by averaging. The statistical procedure of one sample *t*-test was used to determine if the sample of observations could have been generated by a process with the experimental average value. The results obtained are summarized in the table below.

In Table 3, we can see how the node spends most of the time in "sleep," mode with a consumption of 0.036 mA. This consumption increases up to 9.4 mA while the microcontroller is in active mode, acquiring data through the sensors. Finally, while the information is sent through the LoRa module, the consumption is 22 mA for approximately one second.

From the data shown in Table 3, we can calculate the average device consumption for this time interval between data collection as follows:

$$Consumption=\frac{9.4\;\mathrm{mA}\times 4\;s+22\;\mathrm{mA}\times 1\;s+0.036\;\mathrm{mA}\times 88\;s}{93\;s}=0.674\;\mathrm{mA}$$

Once the average consumption of the node is obtained, and knowing the capacity of the battery, we can calculate its theoretical duration:

$$Duration=\frac{2000\;\mathrm{mAh}}{0.674\;\mathrm{mA}}=2967\;h=123\;\mathrm{days}$$

As will be seen later, this theoretical duration is only useful for performing a first estimate. Normally, the actual duration of the battery is well below the theoretically calculated value.

The second test had as its objectives obtaining the discharge curve of the battery and its real duration. Thus, measurements of the voltage supplied by the battery were taken periodically during the operation of the device. In this way, we were able to estimate its state of charge, as well as the time for which the device can operate without needing maintenance or battery change. Next, the voltage values measured against the number of days that it was in operation were plotted on a graph.

**Figure 5:**
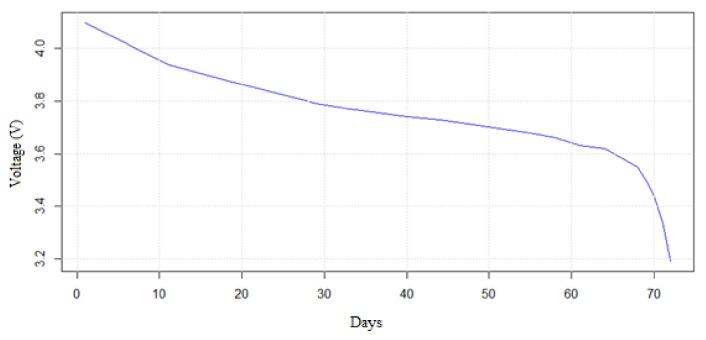
Node battery voltage.

As can be seen in Figure 5, the voltage supplied by the battery starts at 4.1 V and decreases rapidly during the first few days. Meanwhile, as we get closer to the nominal voltage of the battery (3.7 V), the curve decreases more slowly toward this value. Once the voltage drops below the nominal value of the battery, it drops drastically. This behavior is typical of lithium batteries, as is the case here.

During the experiment, the node ran for a total of 72 days, during which it took measurements every 93 s. In this time period, more than 49,000 measurements were taken.

If we compare the battery life obtained in the real experiment with the theoretically calculated one, we can verify that its real life is approximately 60% of the theoretical one. These data may vary depending on the characteristics or battery and environmental conditions. For example, in [1], a value of 70% is taken to estimate the duration of the battery. In contrast, [6,13,38] did not use any correction factor to calculate battery life.

### 4.2. Field Test

**Figure 6:**
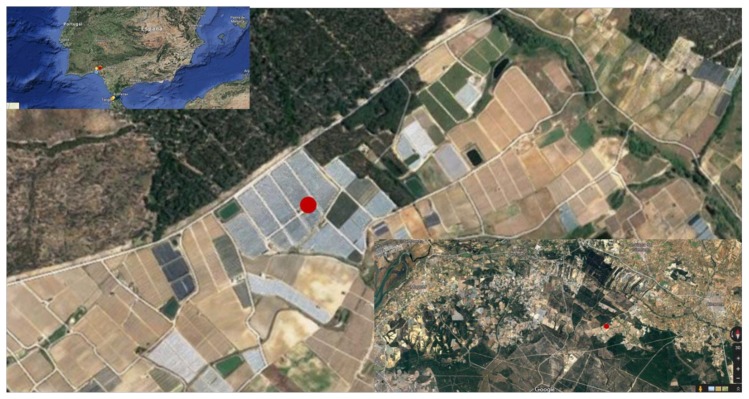
Experimental site for the field test.

The farm selected as a case study is a berry farm located in Huelva (Andalusia, Spain), one of the most important berry producing areas in Europe and where 100% of the crop is irrigated under plastic (Figure 6). In climatic terms, this is a semi-arid zone with an annual rainfall of approximately 630 mm. The validation tests were carried out on a blueberry crop that covers two plots of approximately 4 hectares each.

**Figure 7:**
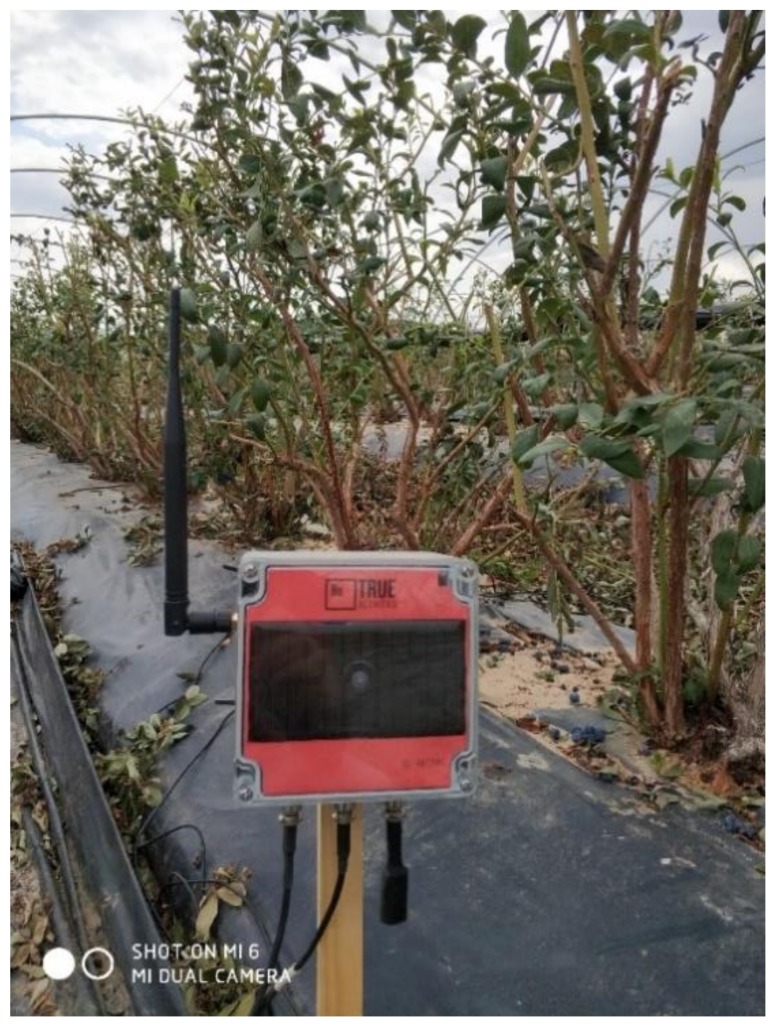
Installed device taking measurements every 30 min.

**Figure 8:**
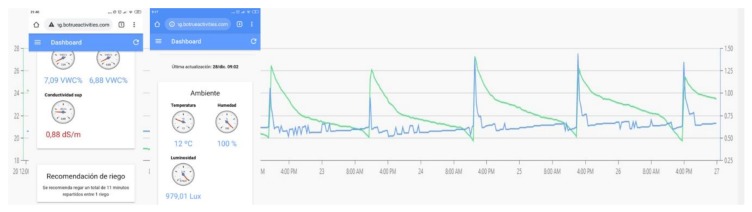
User interface of the application web.

The devices were installed in this crop for 6 months (January–June, 2018) (Figure 7). Figure 8 shows the snapshots of real data from the sensors.

Because the parameters that are be measured in the crop do not show large variations in short time intervals, it was decided that if measurements are taken every 30 min, a correct balance between battery life and adequate precision would be obtained, allowing us to evaluate the state of the crop in real time. Thus, if we use the data previously measured in the different operating modes, we can calculate the average of the device for a time between measures of 30 min in the following way:

$$Consumption=\frac{9.4\;\mathrm{mA}\times 4\;s+22\;\mathrm{mA}\times 1\;s+0.036\;\mathrm{mA}\times 1795\;s}{1800\;s}=0.069\;\mathrm{mA}$$

Taking into account the calculations performed previously, and assuming that the actual duration is 60% of the theoretical calculation, the estimated duration of the battery for a time between data collection of 30 min would be the following:

$$Duration=\frac{2000\;\mathrm{mAh}\times 0.6}{0.069\;\mathrm{mA}}=17391\;h=724\;\mathrm{days}$$

With this autonomy, the end-device would be able to operate for two years without the need for battery change or any maintenance.

## 5. Discussion

Estimating the energy efficiency for an IoT device is very difficult when there are several communication technologies, electronic components, and measuring devices, as well as a multitude of environmental conditions and particular situations for each crop and its needs. These limitations are commented upon in some works [40].

It was not the objective of this work to modify hardware and software parameters to reduce consumption, but to decide on the most appropriate technology and components that would allow us to develop an end-device with low consumption. We also decided on the best intervals for data collection and sending, and the microcontroller was programmed to achieve minimal consumption. Finally, we compared the results with other similar works in the field of smart irrigation.

The results achieved in this study improve upon the recently published ones. For instance, [13] present a node formed by the Msp430F1611 Texas Instruments processor and wireless communication at 2.4 Ghz with a gateway. To measure the different terrain parameters, they used two Hydra Probe II sensors. The measurements were taken every 30 min, obtaining an average consumption of 0.5035 mA. The calculated autonomy was 223 days, achieved by installing a 2700 mAh battery.

In [1], a device using LoRa communication was developed to measure temperature and humidity parameters and soil data. With a time between measures of 60 min, the average consumption of the device was 0.4026 mA, achieving a range of 73 days for a 2400 mAh battery. Unlike the previous case, the author corrected the duration of the battery by the factor 0.75, thus achieving a result closer to reality.

The GAIA2 system [5] communicates via radio at 2.4 GHz with a gateway. In laboratory tests, with a node and a humidity and temperature sensor SHT71, an average consumption of 0.089 mA was obtained. On the other hand, a real field experiment was carried out in which two MPS-2 sensors were used, capable of measuring the water potential and soil temperature, obtaining a total battery life of 56 days.

Finally, the LoRa irrigation system proposed by [19], which uses a lithium battery with 4800 mAh, consumes, in their different operative modes, 2.0987 mA on average, equating to a battery life of 57 days under the same conditions as our experiment.

We presented a system capable of an average consumption of 0.069 mA, thus providing a battery life of up to 724 days. If we compare these results with those obtained in previous articles, we can see that consumption has been reduced significantly, along with a significant increase in battery life.

The following Table 4 shows a comparison of the results obtained in other studies.

If we compare the results obtained in our study with those obtained by [13], we can see that both devices take measurements every 30 min and have two sensors that measure various parameters of the soil. However, the average consumption obtained by our device is a reduction of 86% compared to the consumption of the other device. Regarding the life of the battery, our device is capable of increasing the life of the battery by more than 300% compared to the work in [13].

Comparing the results of our BoXmote with those obtained by [1], we can see that our average consumption is 82% less than that of their device. Bearing in mind that in this case the time between measurements is 60 min, this would mean that average consumption would be even higher if the time were reduced to 30 min. Regarding the life of the battery, the BoXmote has an autonomy 990% greater than the device presented by [1].

Finally, if we compare the results of our BoXmote with [5], we can see that both devices have two soil sensors and a time between measurements of 30 min. In this case, the life of the battery is increased by 1292%, which represents a significant improvement over the solution proposed by [5].

As can be seen, a significant increase in battery life was obtained in terms of wireless sensor networks. In this way, one of the great problems that these devices present during their operation is solved.

Another advantage that our system has over other similar ones that are currently marketed is its low cost. The BoXmote has a cost of 70.00€ compared to the usual cost of end-devices around 350.00€. This low cost allows us to distribute a large number of nodes for the crop, thus obtaining much more precise information.

## 6. Conclusions

Most of the papers related to WSN do not include quantitative results about the impact of the WSN’s deployment in the field [41]. In this paper, we presented a low-cost system based on LoRa technology capable of taking measurements of the parameters most used in precision agriculture, such as humidity and ambient temperature, light received by the plant, and soil temperature, soil moisture, and EC.

The results obtained show better consumption than that of commercial wireless sensors. The consumption of the node was measured in its different operating states, and the battery discharge curve was obtained, achieving, if measurements were taken every 30 min, an approximate battery duration of 724 days. All the consumption required by data acquisition and sending to the gateway was taken into consideration.

In order to increase the battery life and take into account the discharge effect, a solar panel could be installed in the node and evaluation performed on how it affects the consumption of the battery.

Another line of work is related to the type of battery installed. In this case, the operation of the node can be compared with another type of power, such as alkaline or lithium-ion batteries.

We are aware that more work is needed on energy consumption, both from a theoretical and practical point of view. The analysis of gateway consumption is another interesting topic for agriculture, especially in remote areas or developing regions.

Finally, a more detailed study on the evolution of NB-IoT technologies versus LoRaWAN and SigFox should be analyzed.

## Figures and Tables

**Table 1:** Comparison between the different communication technologies for end-devices.

Parameters	ZigBee	Global System for Mobile (GSM)/General Packet Radio Service (GPRS)	Long-Range (LoRa)	SigFox	NarrowBand IoT (NB-IoT)
Standard	ZigBee Alliance IEEE 802.15.4	N/A	Lora Alliance IEEE 802.15.4	IEEE 802.15.4	3GPP
Frequency band	Unlicensed ISM bands 868/915/433 MHz and 2.4 GHz	Licensed bands 900–1800 MHz	Unlicensed ISM bands 868/915/433 MHz	Unlicensed ISM bands 868/915/433 MHz	Licensed LTE bands 700, 800, 900 MHz
Power consumption	Low	Medium	Very Low	Very Low	Medium
Data rate	20, 40, and 250 kbps	Up to 170 kbps	50 kbps	100 bps	200 kbps
Latency	20–30 ms	<1 s	N/A	N/A	Very Low
Communication range	100 m (open spaces)	1–10 km	20 km (rural)	40 km (rural)	10 km (rural)
End-device cost	Low	Medium	Low	Low	Medium
Network Topologies	P2P, tree, star, mesh	Cellular system	Star of stars	Star	Cellular system
Limitations	Line-of-sight between the sensor node and the coordinator node must be available	Power consumption	Network size (scalability), data rate, and message capacity	Low data rates	Lower range and coverage capabilities (i.e., range <10 km)

**Table 2:** Popular wireless nodes used in the agriculture domain.

Sensor Name	Parameters Captured	Company
ECH2O	Soil moisture, Soil temperature, Electrical conductivity	Meter Group, Inc, Pullman, WA, USA
HydraProbe	Soil moisture, Soil temperature, Electrical conductivity	Stevens Water Monitoring Systems, Inc, Portland, OR, USA
MP406	Soil moisture, Soil temperature	ICT International, Armidale, Australia

**Table 3:** Average power consumption (mA) for the different modes.

MODE	CONSUMPTION (mA)	TIME (s)	% TOTAL TIME
Active	9.4	4	4.30%
Sending	22.0	1	1.07%
Sleep	0.036	88	94.62%

**Table 4:** Comparison of data obtained with other methods.

Method	Technology Tested in Smart Irrigation	Average Consumption	Time between Measures	Sensors	Battery Life Specified in Each Paper
Our BoXmote	LoRa/2200 mAh battery	0.0690 mA	30 min	1 × ECH2O 10HS, 1 × ECH2O 5TE, 1 × DHT11, 1 × light-dependent resistance	724 days
[1]	LoRa/2400 mAh battery	0.4026 mA	60 min	1 × TCN75A digital temperature sensor, 1 × Texas Instruments HDC1050 Low Power Digital Humidity Sensor, 1 × photo-resistance, 1 × own soil moisture sensor	73 days
[19]	LoRa/SX1276 transceiver STM32L151CB Microcontroller/4800 mAh battery	2.0987 mA	30 min		57 days
[13]	ZigBee ED (Open-ZB)/CC2420 module/Msp430F1611 processor/2700 mAh battery	0.5035 mA	30 min	2 × Stevens Hydra Probe II soil moisture sensor	223 days
[5]	ZigBee ED (Open-ZB)/CC2420 module/Msp430F1611 processor/2700 mAh battery	Not specified	30 min	2 × MPS-2 soil water potential sensor	56 days
[6]	GPRS/1900 mAh battery	5.93 mA	15 min	2 × MPS-2, 1 × Hydra Probe II	13.35 days
